# Anti-Cancer Activity of a Novel Small Molecule Compound That Simultaneously Activates p53 and Inhibits NF-κB Signaling

**DOI:** 10.1371/journal.pone.0044259

**Published:** 2012-09-13

**Authors:** Sun Gwan Hwang, Jinah Park, Joo Young Park, Cheol Hyoung Park, Ki-Ho Lee, Jeong Woo Cho, Jong-Ik Hwang, Jae Young Seong

**Affiliations:** 1 Drug Development Center, SK Biopharmaceuticals Co., Ltd., Daejeon, Korea; 2 Laboratory of G Protein Coupled Receptors, Graduate School of Medicine Korea University, Seoul, Korea; 3 Korean Bioinformation Center, KRIBB, Daejeon, Korea; Wayne State University School of Medicine, United States of America

## Abstract

The p53 and NF-κB pathways play important roles in diverse cellular functions, including cell growth, apoptosis, and tumorigenesis. Mutations that inactivate the p53 gene and constitutive NF-κB pathway activation are common occurrences in human cancers. Although many drugs are being developed that selectively activate p53 or inhibit NF-κB, there are few drug candidates that can do both. Simultaneous activation of p53 and inhibition of the NF-κB pathway is therefore a prime target for new cancer drug development. This study is the first report of a high-throughput approach with mass compounds that concurrently target both pathways. Using a cell-based screening assay and a library of 200,000 synthetic compounds, we identified 9 small molecules that simultaneously inhibit NF-κB and activate p53. One of these compounds, N-2, increased the expression of p53 target genes, including p21 and GADD45a. In addition, N-2 inhibited the transcriptional activity of NF-κB, concomitantly repressing interleukin-6 and monocyte chemotactic protein-1 (MCP-1) expression. When cell lines derived from a diverse range of cancers were treated *in vitro* with N-2, we observed increased cell death. N-2 also significantly inhibited allograft growth in murine models of melanoma and lung carcinoma. Our findings suggest that N-2 may act as a bivalent anti-cancer agent through simultaneous modulation of NF-κB and p53 activities.

## Introduction

The NF-κB and p53 signaling pathways function in nearly all cell types and are activated in response to numerous biological stimuli. NF-κB is a key player in diverse cellular functions [1], [2]. Although first identified as a transcription factor involved in the inflammatory response, experimental evidence suggests that NF-κB also regulates cell growth, survival, and apoptosis [3]. IκB proteins inhibit NF-κB function by preventing NF-κB from binding DNA. Activation of NF-κB involves phosphorylation of specific IκB serine residues by IκB kinases (IKKs) leading to proteasome-mediated degradation of IκB. Upon IκB degradation, the NF-κB complex is then free to enter the nucleus where it can regulate the expression of specific genes related to inflammatory or immune responses, cell survival responses, and cellular proliferation [4]. The tumor suppressor protein p53 is a DNA binding transcription factor that plays an important role in guarding the cell in response to various stress signals [5]. Activated p53 induces expression of several genes related to cell cycle arrest, apoptosis, senescence, translation, and DNA repair. Phosphorylation of p53 at particular serine residues involves its own activity. For instance, phosphorylation of serines 9 and 46 is related to the induction of apoptosis and DNA damage [6], [7]. Phosphorylation at serines 15 and 20 leads to a reduced interaction with its negative regulator, murine double minute 2 (MDM2). MDM2 inhibits p53 accumulation by targeting it for proteasome-mediated degradation [8], [9].

Constitutive activation of NF-κB is frequently observed in human cancers of diverse origins, including lung, melanoma, and colorectal cancer, and it is associated with angiogenesis, chemotherapy resistance, and survival of cancer stem cells [10], [11], [12], [13]. Tumor-cell-associated NF-κB and its regulated genes, such as the cytokine IL-6, have been linked to the development of chemoresistance in several types of cancers [14], [15]. For example, IL-6 is elevated in the serum and ascites of patients with ovarian cancer and increased IL-6 concentrations correlate with poor prognoses and chemoresistance [16]. Such resistance to chemotherapy can severely affect the efficacy of anti-cancer agents. The NF-κB pathway has gained more attention as an emerging therapeutic target in cancer cells harboring mutations in the Ras gene family, one of the most frequently mutated gene families in human cancers. It is known that approximately 20 to 30% of non-small-cell lung cancer patients (approximately 85% of all lung cancers) have oncogenic mutations in k-Ras [17]. Inhibition of NF-κB signaling impairs cellular transformation and sensitizes Ras-mutated cancer cells to undergo apoptosis [11], [18], [19], [20], [21]. This inhibition might therefore be a promising strategy for treating tumors that have Ras mutations and other cancers that express constitutively active Ras.

Mutations in the p53 gene are more common in tumors than mutations in the Ras gene family. In fact, p53 is directly mutated in approximately 50% of human tumors [22]. Restoring p53 function may therefore provide an attractive therapeutic strategy to target cancer cells and thus, small molecules such as the MDM2 antagonist Nutlin-3 [23], the p53-binding molecule RITA [24], and the MDM2 down-regulator gambogic acid [25], have been developed. However, restoration of p53 function is not sufficient for complete tumor cell loss. For example, p53 overexpression had no effect on the development of low-grade lesions such as adenomas and p53 does not cause complete tumor cell loss in high-grade lesions such as carcinomas [26], [27], [28].

Many studies have investigated the role of the NF-κB and p53 pathways under pathological conditions, particularly cancer [4], [29]. Activation of the NF-κB pathway and inactivation of the p53 pathway are detected in diverse cancers. However, it is unlikely that an agent targeting just one of these pathways may be effective for treating various types of cancer, given the complexity of tumorigenesis. Several p53-inducing chemotherapeutics have been reported to induce p53 as well as NF-κB in different types of cells [30]. Despite p53-induced apoptosis, NF-κB activation is able to promote resistance to apoptosis. Constitutive NF-κB activation has been reported in diverse human cancers and is also related to drug resistance [11].

These findings strongly suggest that compounds with the ability to repress NF-κB pathway function while also activating the p53 pathway might possess higher anti-cancer efficacy. Indeed, the cyclin dependent kinase (CDK) inhibitor seliciclib (r-roscovitine) [31] and the malaria drug quinacrine (QC), a 9-aminoacridine (9AA)-derivative [32], possess this dual activity and are currently in phase II clinical trials as an anti-cancer agents. QC and seliciclib are reported to inhibit NF-κB via inhibition of Ser-536 phosphorylation of the p65 subunit of NF-κB [31], [32]. Ser-536 phosphorylation of the p65 subunit is essential for NF-κB activity and dephosphorylation of this site converts NF-κB into a non-active form. In addition to this activity, QC is able to activate the p53 pathway and induce tumor cell death through Bcl-2-associated X protein (BAX) [33] and seliciclib activate p53 by inhibiting MDM2-mediated p53 degradation [34]. Therefore, these drugs present a promising opportunity to treat cancers in which both of these pathways are deregulated and Ras is constitutively active.

In the present study, we identified small molecule compounds that simultaneously activated p53 and inhibited NF-κB using a forward chemical genetic approach. Of these compounds, N-2 induced the death of diverse cancer cell types via inhibition of NF-κB and induction of p53. N-2 also inhibited tumor growth in the B16F10 melanoma and LLC lung carcinoma murine allograft models. Thus, N-2 might represent a potential chemotherapeutic drug with the ability to target a diverse range of malignant tumors.

## Results

### Identification of small molecules that simultaneously activate p53 and inhibit NF-κB

**Figure 1:**
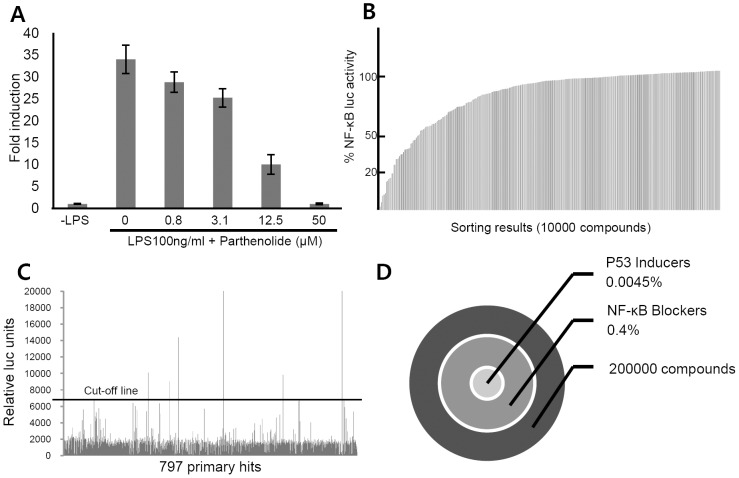
Identification of small molecules that simultaneously activate p53 and inhibit NF-κB. C6 cells derived from rat glioma carrying wild type p53 were used to screen the library. (**A**) The dose-dependent response of the NF-κB reporter gene with increasing concentrations of parthenolide. (**B**) Screening of compounds that inhibit the NF-κB reporter gene using the library of 200,000 compounds. Compounds that inhibited 100 ng/ml LPS-induced reporter activity more than 80% were selected as primary hits (n = 797). (**C**) Screening of the primary hit compounds for activation of the p53 reporter gene. Compounds that activated the p53 reporter more than 3-fold were selected as the final hits (n = 9). (**D**) Summary of HTS procedures. The total hit ratio was approximately 0.0045%.

The library was screened to identify compounds that simultaneously modulate the p53 and NF-κB pathways. To assess the sensitivity and robustness of the cell-based assay before performing HTS, we confirmed dose-response profiles in the HTS format using the positive control parthenolide that blocks LPS-induced NF-κB activity (Fig. 1A). The IC_50_ value for parthenolide was 7.2 µM, which is in agreement with a previous report [35], [36]. The optimization and miniaturization of HTS were performed to achieve 384-well plate formats. The Z′ values of the cell culture plates were >0.6.

The first round of HTS using the NF-κB reporter gene identified 797 primary hits, equivalent to a 0.4% hit ratio (Fig. 1B). A second HTS was then performed to assess the ability of these 797 compounds to activate the p53 reporter gene. This screen identified 9 compounds that satisfied the study criteria (Fig. 1C). The total hit ratio of the screening process was 0.0045% (Fig. 1D).

### Classification of bivalent small molecule compounds

**Figure 2:**
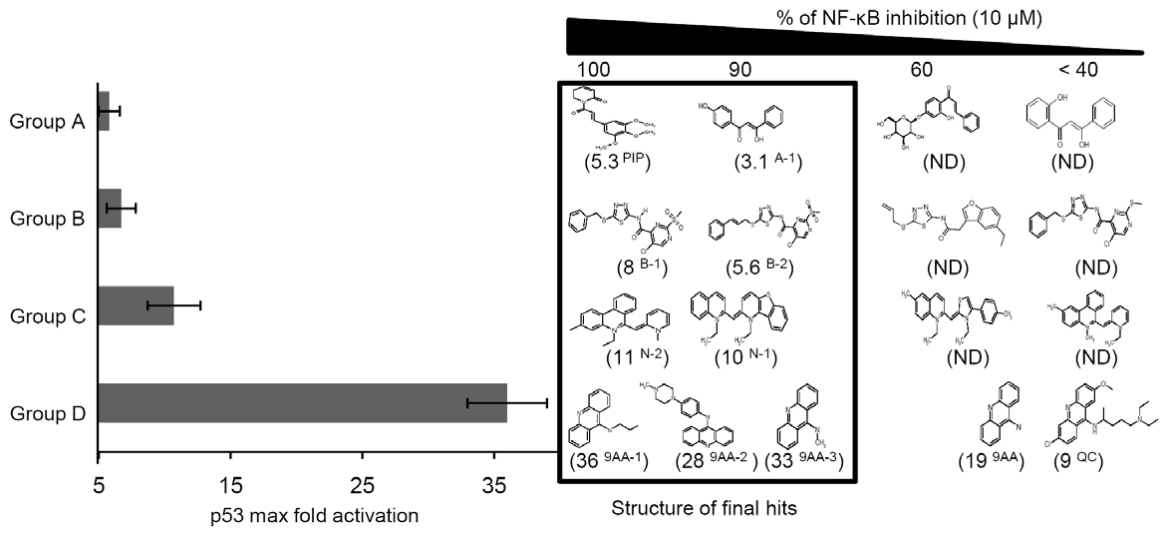
Classification of the bivalent molecules identified by HTS. Compounds that induced the p53 reporter more than 3-fold and simultaneously inhibited the NF-κB reporter by more than 80% were included in the analyses (n = 9). In addition, 8 less active derivatives of similar structure were included for structure: activity relationship evaluation. Values in parentheses refer to maximum p53-responsive reporter induction in cells treated for 8 h with each compound (0.4–25 µM). Relative NF-κB inhibition is presented as the percentage inhibition of the NF-κB reporter in the presence of 10 µM of each compound. The 17 compounds were grouped according to structural similarity (Groups A–D).

The final 9 compounds identified following two rounds of HTS were grouped by their chemical structure (Fig. 2). Group D included 9AA-derivatives, such as 9AA and QC. The 9AA-derivatives 9AA-1 and 9AA-2 have been reported to activate p53 but inhibition of NF-κB has not been reported [33], [37]. Half of the hit compounds were 9AA derivatives and these compounds showed good correlation between NF-κB inhibition potential and p53 activation (R^2^ = 0.85, Supplementary Figs. S1A and B).

The Group A compound piperlongumine (PIP) has been reported to selectively kill cancer cells based on cancer-specific features and this compound was previously screened using a p53-responsive reporter assay in U2OS osteosarcoma cells [38]. Interestingly, in our screen, we found that PIP not only activates p53 but also is a strong inhibitor of NF-κB activity (Fig. 2).

Among novel hit compounds, N-2 (5-Ehyl-3-methyl-6-(1-methly-1H-pryidin-2-ylidenemethyl)-phenanthridinium, C_23_H_23_N_2_, M.W. = 327.5) showed the most promising profile. N-2 induced the p53 reporter more than 10-fold and strongly inhibited NF-κB activity compared with other novel hits in Groups A through D. Therefore, we chose this compound for further analyses. QC, which is currently in clinical trials for the treatment of hormone-refractory taxane-resistant prostate cancer, and 9AA were selected as control compounds.

### N-2 inhibits NF-κB and its downstream target genes IL-6, MCP-1, and nitric oxide

**Figure 3:**
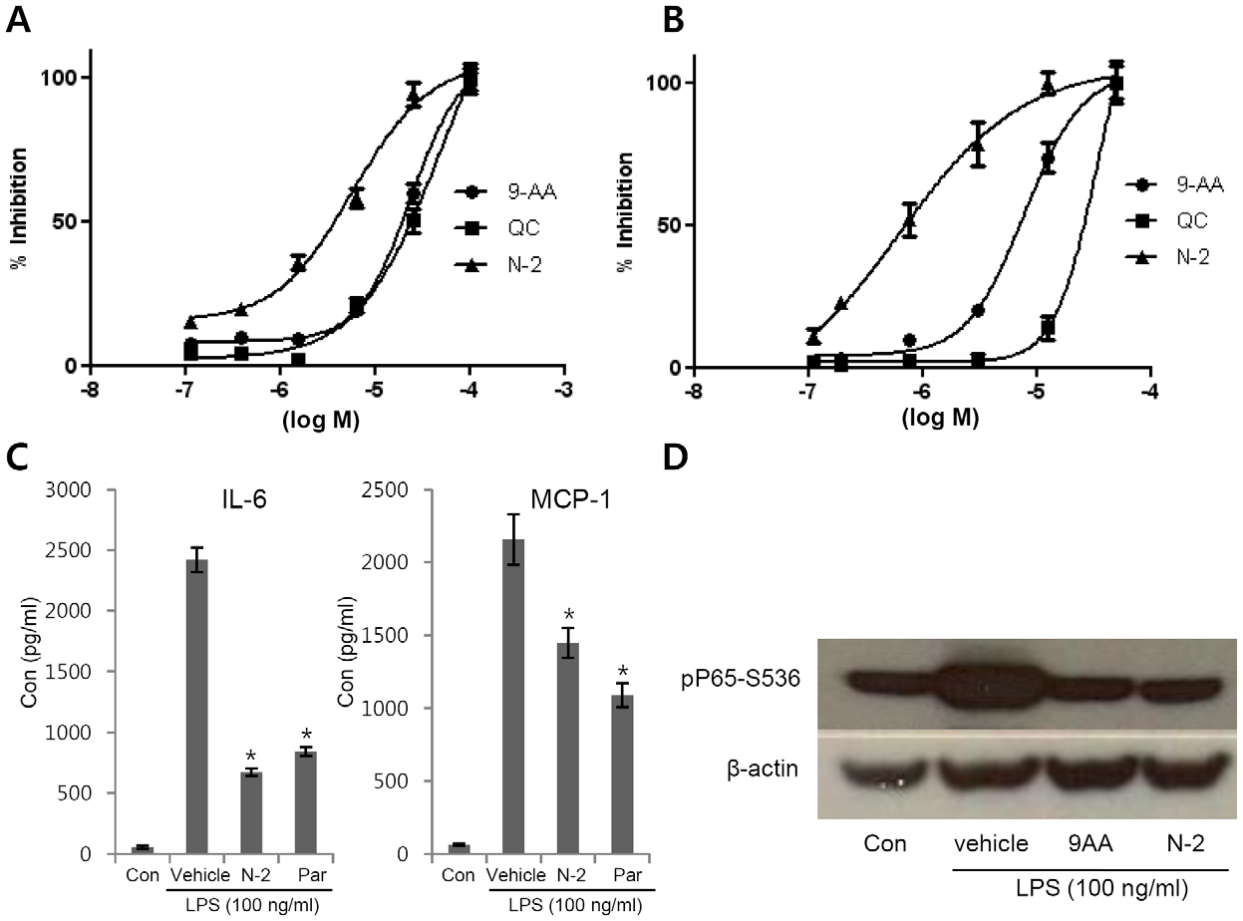
Effect of N-2 on LPS-stimulated gene expression and serine phosphorylation of NF-κB. (**A**) The dose dependence of NF-κB reporter gene luciferase activity was determined in C6 cells with increasing concentrations of 9AA, QC, and N-2 (mean ± SD of three independent experiments). (**B**) The dose-dependent inhibition of nitric oxide (NO) production was determined with increasing concentrations of 9AA, QC, and N-2 in RAW 264.7 cells. The results shown are the mean ± SD of three experiments. (**C**) Levels of IL-6 and MCP-1 in RAW 264.7 cells following treatment with parthenolide (Par) or N-2 in the presence of LPS. Each data point represents the mean ± SD of four assays. * p<0.01 by paired Student's t-test. (**D**) Analysis of Ser536 phosphorylation of the p65 subunit of NF-κB in RAW 264.7 cells following treatment for 1 h with 20 µM of QC or 1 µM of N-2 in the presence of LPS. β-actin was used as a loading control.

Compared with 9AA and QC, N-2 showed increased inhibition of the NF-κB reporter in C6 cells when added simultaneously with 100 ng/ml LPS. The IC_50_ values of 9AA, QC, and N-2 for the NF-κB reporter gene were 23.8 µM, 50.4 µM, and 5.9 µM, respectively (Fig. 3A). LPS stimulates NF-κB-mediated nitric oxide production as well as expression of cytokines and chemokines, such as IL-6 and MCP-1 [39]. Treatment of murine RAW 264.7 macrophage cells with N-2 strongly inhibited LPS-induced nitric oxide production; the IC_50_ values of nitric oxide production by 9AA, QC, and N-2 were 7.8 µM, 33.7 µM, and 0.64 µM, respectively (Fig. 3B). We also evaluated inhibition of LPS-induced IL-6 and MCP-1 expression in RAW 264.7 cells. N-2 and the positive control parthenolide significantly inhibited IL-6 and MCP-1 protein production (Fig. 3C). Treatment with N-2 (1 µM) inhibited LPS-induced phosphorylation of Ser-536 of the p65 subunit of NF-κB, similar to 20 µM of 9AA (Fig. 3D). Together, N-2 strongly inhibited the NF-κB reporter and its downstream cytokine IL-6 (Figs. 3B and C), a property that provides a rationale for chemoresistant cancer therapy using N-2.

### N-2 activates p53 in cancer cells of diverse origin

**Figure 4:**
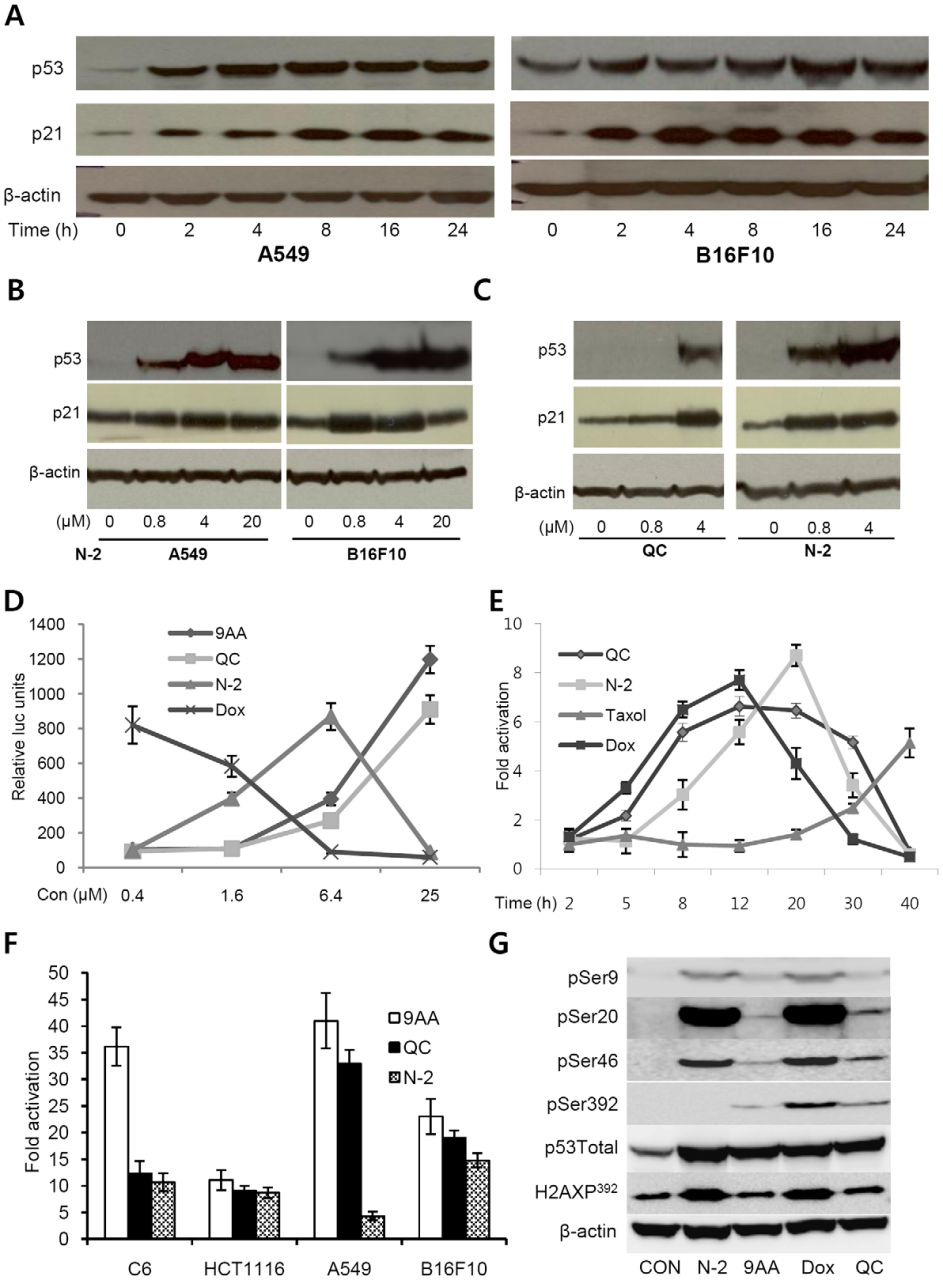
Effect of N-2 on p53 and expression of its target gene p21. (**A**) The levels of endogenous p53 and p21 proteins were evaluated following treatment of A549 and B16F10 cells with 1 µM of N-2. (**B**) Dose dependency of N-2 on endogenous p53 and p21 proteins. (C) Dose dependency of N-2 and QC on endogenous p53 and p21 proteins in HCT116 cells. (D) The p53-responsive reporter activity in HCT116 cells treated for 12 h with 9AA, QC, doxorubicin (Dox) or N-2 over a range of concentrations (0.4–25.0 µM). (**E**) Activation kinetics of the p53-responsive reporter activity in HCT116 cells treated for 2–40 h with 3 µM of N-2, 3 µM of taxol, 1 µM of Dox or 10 µM of QC. (**F**) The p53 reporter activity in C6, HCT116, A549, or B16F10 cells treated for 12 h with 9AA, QC, or N-2 over a range of concentrations (0.4–25 µM). The data are shown as the relative fold induction of the p53 reporter gene at the most effective concentration of each compound. The results shown are the average of three experiments; the bars indicate standard deviation (D–F). (**G**) Phosphorylation of p53 at various serine residues and phosphorylated histone γ-H2AX in A549 cells treated for 12 h with QC (10 µM), 9AA (5 µM), Dox (1 µM) or N-2 (1 µM).

N-2 increased total endogenous p53 protein expression and expression of its target gene p21 in A549 human lung cancer cells and B16F10 murine melanoma cells in a time- and dose-dependent manner (Figs. 4A and 4B). Activation of endogenous p53 and p21 reached maximum levels 8 h after treatment with N-2 in A549 cells and 16 h in B16F10 cells. N-2 induced the p53 reporter gene and its endogenous target gene p21 in HCT116 cells with higher efficacy than QC (Figs. 4C and 4D). Activation of the p53-responsive reporter by 1 µM of N-2 showed similar kinetics to 10 µM of QC or 1 µM Dox (Fig. 4E). The maximum effect of Dox, N-2 and QC on p53 reporter activation was observed at 12 to 20 h after treatment while 3 µM of taxol displayed peak p53 reporter activity after 40 h (Fig. 4E). 9AA, QC, and N-2 activated the p53 reporter gene in diverse cell lines, including C6, HCT116, A549, and B16F10 (Fig. 4F). Maximum p53-responsive reporter activity was observed in cells treated with each compound with range of 0.4–25 µM. (Fig. 4F). No increase in p53 mRNA expression was found in response to N-2 treatment (data not shown). Instead, treatment of A549 cells with 1 µM N-2 for 12 h induced p53 phosphorylation at Ser9, Ser20, and Ser46 (Fig. 4G). However, N-2 was not able to induce phosphorylation of Ser-392 that was phosphorylated after 9AA or QC treatment. To evaluate whether N-2 causes DNA damage, we examined phosphorylation of γ-H2AX at Ser139. N-2 and Dox increased levels of phosphorylated γ-H2AX (Fig. 4G and Supplementary Fig. S2). We found no such activity by 9AA and QC as previously reported [32]. Therefore, it is likely that N-2 stabilizes p53 via DNA damage-mediated phosphorylation of p53.

### N-2 has anti-tumor effects *in vivo*

**Figure 5:**
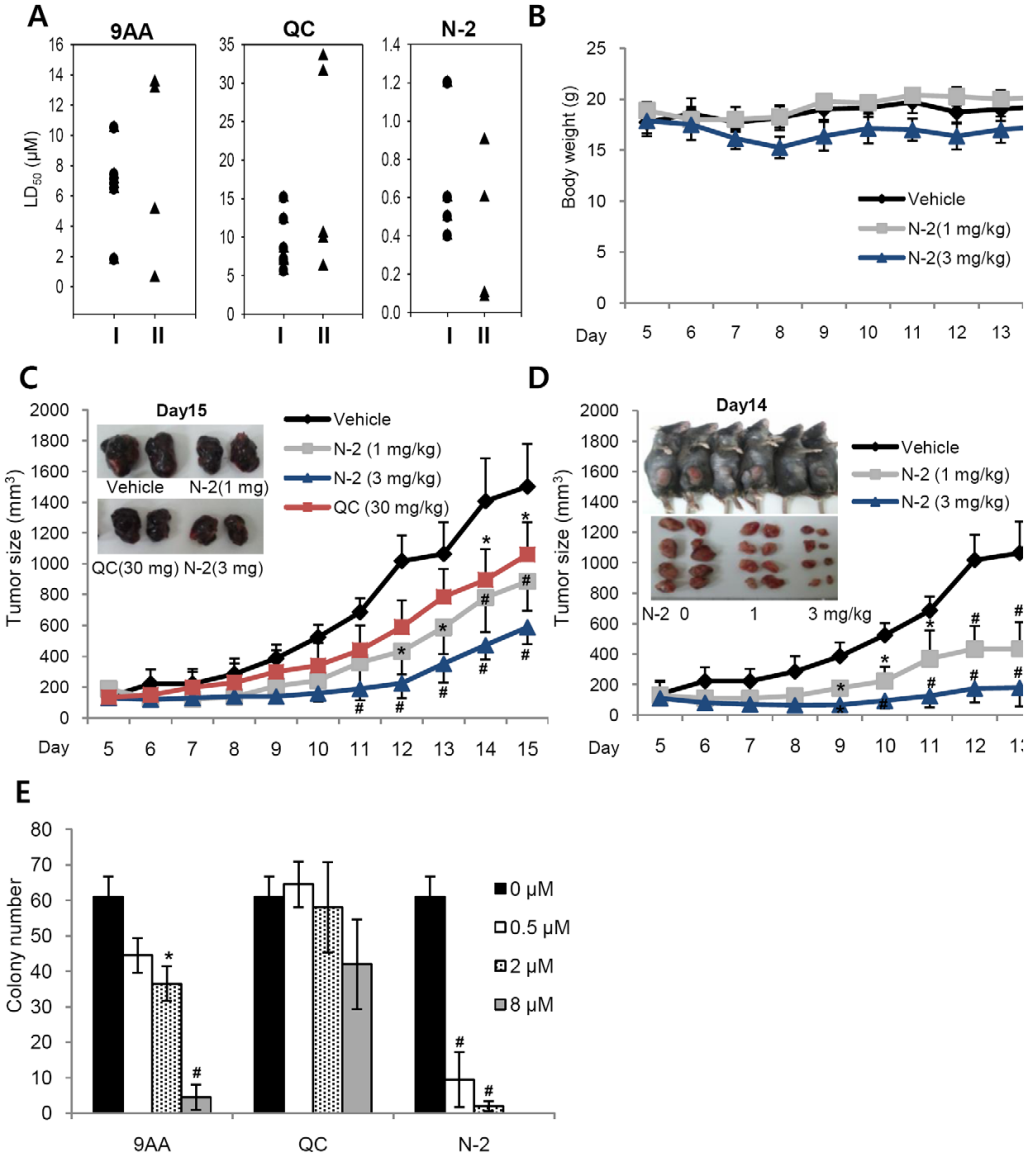
Effect of N-2 on diverse tumor cell line growth *in vitro* and *in vivo*. (**A**) Comparison of the IC_50_ concentrations of 9AA, QC, and N-2 in different wild-type and mutant p53 cell lines. Each point represents the IC_50_ of a particular type of cell, which are grouped as follows: (*i*) circles, p53 wild-type cell lines (A549, H460, HCT116, C6, SH-SY5Y, and B16F19); and (*ii*) triangles, p53 mutant cell lines (H2009, SW480, Jurkat, U937, and LLC). (**B**) Time course of body weight changes following treatment with N2. Error bars represent the SD. (**C**) The anti-tumor activity of N-2 and QC on B16F10 allografts. Tumor volume and a representative excised melanoma at day 15 are shown. (**D**) The anti-tumor activity of N-2 on LLC allografts. The tumor volume, appearance of allografts, and excised LLC tumors at day 14 are shown. Error bars represent the SD (C–D). * indicates significance compared with the control, p<0.05, ^#^ p<0.01 with ANOVA. (E) Effect of N-2 anchorage-independent growth in A549 cells. Each data point represents the mean ± SD of three assays. *p<0.05, ^#^ p<0.01 by paired Student's t-test.

We next compared the anti-proliferative activity of 9AA, QC, and N-2 on different types of cancer cells. The LD_50_ concentration for each cell type was determined after treatment for 48 h with 9AA, QC, and N-2 in various wild-type and mutant p53 cells. N-2 showed relatively higher efficacy than 9AA and QC in all cell lines tested (Fig. 5A). Particularly, N-2 showed the most potent efficacy in LLC cells (LD_50_ = 80 nM). Therefore, we selected LLC cells for further *in vivo* tests. In addition, we examined *in vivo* effect of N-2 on mouse B16F10 melanoma cells (LD_50_ = 600 nM) as melanoma and lung cancer are two of the most drug-resistant tumor types.

We assessed whether N-2 had anti-tumor effects in mouse B16F10 (melanoma) and LLC allograft models. Activators of p53 have shown promise in treating primary uveal melanoma and murine allograft models of ocular B16F10 cell melanoma [40]. QC and N-2 inhibited the growth of tumor allografts formed by intraperitoneal injection of B16F10 melanoma cells in C57/BL6 mice. N-2 (1 mg/kg) showed similar anti-tumor effects as 30 mg/kg QC without significant weight loss (up to 20% at 3 mg/kg treatment of N-2; Figs. 5B and C). Additionally, N-2 showed strong efficacy in an *in vivo* LLC allograft model (Fig. 5D). The results of the LLC allograft correspond with the most potent *in vitro* LD_50_ value of N-2 in LLC cells. The mean weight and volume of the primary tumors in N-2-treated mice were significantly lower than the vehicle-treated mice.

Inhibition of NF-κB signaling induced apoptosis in p53-null lung cancer cells and inhibited mouse lung adenocarcinoma development [19], [41], [42]. To evaluate the effect of N-2 on human lung cancer, we used agar colony formation assays. NF-κB plays an important role in anchorage-independent growth, metastasis, and tumor formation in lung carcinoma cells, including A549 cells [43]. Colony formation *in vitro* generally correlates with tumorigenicity *in vivo* [44]. N-2 strongly inhibited colony formation in a dose-dependent manner and its efficacy is much higher than 9AA and QC in A549 colony formation assays (Fig. 5E).

### N-2 induces stress response genes

**Figure 6:**
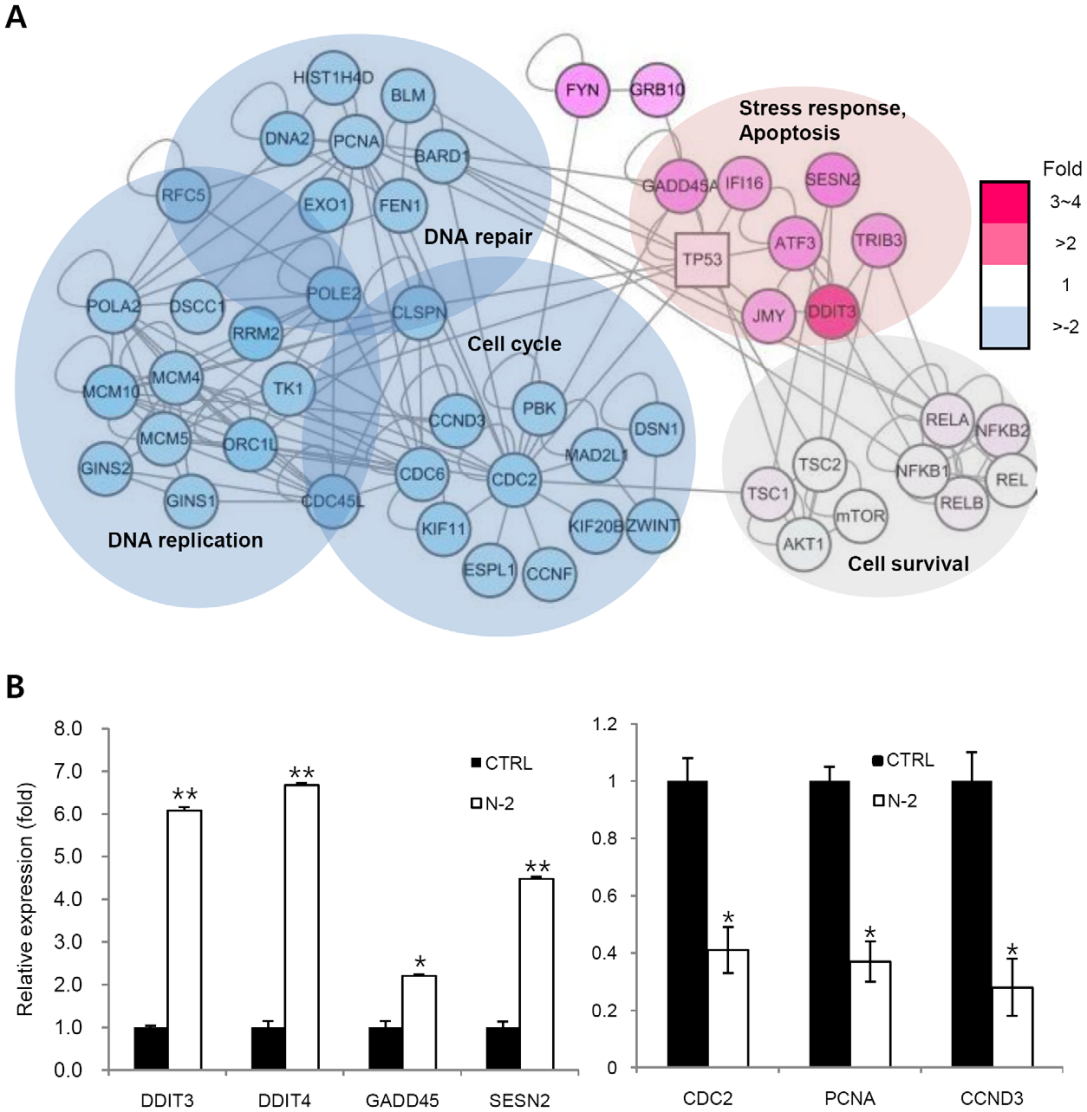
Global expression profiling of N-2-responsive genes in A549 cells. (A) Interaction map of DEGs. Overall network reveals many interesting interactions that may be connected to p53 or NF-κB. The node color represents fold change (green, downregulation; red, upregulation). (B) N-2 up-regulates the mRNA levels of stress response genes (DDIT3, DDIT4, SESN2, and GADD45a) and down-regulates the mRNA level of genes associated with proliferation (PCNA and CCND3). The transcription level of selected genes was confirmed with RT-PCR. * p<0.01, ** p<0.001.

We visualize molecular interaction network by analyzing relation of the differentially expressed target genes displayed in microarray assay. The overall network reveals several interesting interactions that may be connected to p53 or NF-κB. N-2 treatment decreased the expression of genes related to cell cycle, DNA replication, and repair but increased expression of apoptosis- and cellular stress-related genes (Fig. 6A and Supplementary Table S1 and S2). These results were verified using RT-PCR. N-2 treatment distinctively induced stress-related genes, such as DDIT3, DDIT4, SENS2, and GADD45 with concomitant down-regulation of the proliferative marker PCNA, CDK1 (CDC2), and the NF-κB target gene CCND3 (Cyclin D3; Fig. 6B).

## Discussion

The transcription factors p53 and NF-κB are two critical proteins that are deregulated in various human cancers. A multi-target approach may be the future of anti-cancer therapy in which drug resistance is an issue. In light of this, novel molecules that dually regulate NF-κB and p53 signaling may be particularly important to evaluate the potential therapeutic use of bivalent drugs in diverse cancers [29].

Our screen of 200,000 compounds identified N-2, a bivalent molecule with the potential to modulate both NF-κB and p53 signaling. Compared with the previously reported bi-targeted drugs, 9AA and QC, N-2 showed stronger efficacy in diverse tumor cell lines. The strong efficacy of N-2 is exhibited by the IC_50_ values for the NF-κB-mediated production of nitric oxide by 9AA, QC, and N-2 (7.8 µM, 33.7 µM, and 0.64 µM, respectively). N-2 also induced p53 and its target genes at lower concentrations than QC. p53 activation is also reported to inhibit LPS-induced NF-κB activation [45]. Interestingly, the IC_50_ of N-2 for cancer cells was independent of the p53 genetic status under the same conditions (Supplementary Table S3 and Fig. S4). Curaxins, QC and previously known p53 activator PIP (Group A) also cause p53-independent apoptotic death of cancer cells [46], [47]. Inhibition of NF-κB signaling alone might be effective for treating lung cancers that have Ras mutations [19], [41], [48]. 9AA and QC also showed no significant difference in LD_50_ values between wild-type and mutant p53 cancer cells (Fig. 5A and Supplementary Table S3), indicating that p53-dependent apoptosis is not the only mechanism of N-2-mediated tumor cell death. Particularly, result in Fig. S4 suggests that ROS likely acts as the signal molecules for N-2-induced cell death and this process is still functional even in the absence of p53. Thus, induction of ROS may provide a possible mechanism for N-2 induced cell death in both p53-wild-type and p53-mutant cancer cells.

In this study, we found that the previously known p53 activators PIP, 9AA-1, and 9AA-2 also inhibited LPS-induced NF-κB activation (Fig. 2). The anti-inflammatory property of the previously identified bivalent drug seliciclib (roscovitine) could be explained by inhibition of NF-κB [31]. In future studies, it will be necessary to evaluate the anti-inflammatory efficacy of these hit compounds in greater detail.

The global gene expression profiling results showed a distinctive induction of stress-related genes, such as ATF3, DDIT3, DDIT4, SENS2, and GADD45 (Fig. 6). It has been reported that various cellular stresses, including endoplasmic reticulum, genotoxic, and reactive oxygen species stress, can stabilize the p53 protein. Effective inducer of p53 RITA also stimulated the stress genes such as ATF3, DDIT3, DDIT4 and GADD45 like N-2 [49]. The DDIT3/CHOP gene is a component of the endoplasmic reticulum stress response, DDIT4/REDD1 and SENS2 are inhibitors of the mTOR survival pathway, and GADD45 is a critical stress sensor of apoptotic cell death by chemo-preventive drugs. Furthermore, ATF3, DDIT3, DDIT4, GADD45 and SESN2 are also p53-regulated genes [50], [51], [52], [53]. Particularly, N-2 induced the expression of the mTOR inhibitors DDIT4 and SESN2 and inhibited phosphorylation of Ser536 of the p65 subunit of NF-κB, which is phosphorylated by AKT [54]. The mechanism of N-2 action might involve inhibition of the AKT/mTOR and AKT/NF-κB pathways. mTOR has emerged as a critical growth-control node receiving stimulatory signals from Ras and phosphatidylinositol-3-OH kinase [55]. The results of our global gene expression profiling suggest that the induction of DNA damage, ROS production and up-regulation of stress-related genes by N-2 are likely involved in p53 stabilization, along with inhibition of the AKT/mTOR or AKT/NF-κB survival pathways.

Treatment with N-2 induced p53 phosphorylation at Ser9, Ser20, and Ser46 in A549 cells. Phosphorylation of these sites is related to the induction of apoptosis, DNA damage, and p53 stability [6], [7], [8]. Especially, phosphorylation of p53 at Ser20 mostly occurs in response to DNA damage and ROS induction [56]. We confirmed the DNA damage by N-2 through the induction of histone γ-H2AX phosphorylation. We determined the effect of N-2 on ROS production in A549 cells using 2′,7′-dichlorofluorescein diacetate (DCF-DA). Treatment of N-2 increased ROS levels significantly in A549 cells but 9AA did not. Positive control phorbol myristate acetate also caused an increase of ROS, but N-2- enhanced ROS was much greater than other compounds (Supplementary Fig. S3). Taken together, these data suggest that the strong efficacy of N-2 in diverse cancer cell types is due to the combination of many factors that target two key pathways in tumor cells. The similarities and differences among QC, 9AA, Dox, RITA, Nurtlin-3, β-phenylethyl-isothiocyanate (PEIC) and N-2 were summarized in Supplementary Fig. S5.

In summary, the novel small molecule N-2 is a bivalent anticancer agent that has a stronger efficacy than other previously identified small molecules. N-2 has the potential for treating diverse cancers, including those resistant to current therapies. This study also provides mechanistic insights into the molecular mechanism of action of other known p53 inducers such as PIP, 9AA-1, and 9AA-2 and highlights the possible use of these compounds as anti-inflammatory agents.

## Materials and Methods

### Cell culture, transfections, and luciferase assays

HCT116, C6, B16F10, A549, U937 and Lewis lung carcinoma (LLC) cells were obtained from the American Type Culture Collection and HCT116 null cells were a generous gift from Dr. Bert Vogelstein [57]. H460, H2009, SW480, Jurkat and SH-SY5Y cells were obtained from Korean cell line bank (Seoul, Korea). Cells were maintained in RPMI 1640 media, supplemented with 10% fetal bovine serum (FBS), 120 µg/ml penicillin, and 200 µg/ml streptomycin, and were incubated at 37°C with 5% CO_2_. Transfections were performed with Lipofectamine 2000 (Invitrogen, San Diego, CA, USA) according to the manufacturer's instructions. For stable expression of pNF-κB-Luc and pp53-Luc vectors (Clontech, Palo Alto, CA, USA), the same method as transient transfection was used except the cells were co-transfected with pcDNA3.1(+) at a molar ratio of 10∶1. Stable colonies were selected with 1,000 ng/mL of G418 (Invitrogen, San Diego, CA, USA) and individual colonies were transferred to a 24-well plate. To examine NF-κB-mediated or p53-mediated transcriptional activities, transiently transfected cells and positive clones were evaluated using the Promega luciferase reporter assay system according to the manufacturer's instructions (Promega, West Virginia, USA). Positive clones were maintained in the presence of 500 ng/mL of G418. Luciferase signals were detected using a luminometer (Victor 5, Perkin Elmer Wallac, Waltham, MA, USA).

### Cell-based high-throughput screening (HTS) to identify small molecules that simultaneously activate p53 and inhibit NF-κB

To screen for small molecules that simultaneously modulate the p53 and NF-κB pathways, we generated a C6 reporter cell line (derived from rat glioma carrying wild type p53) stably transfected with p53-responsive and NF-κB-responsive luciferase reporter genes. The compound library consisted of 200,000 chemicals provided by the ChemBridge (San Diego, CA, USA), ChemDiv (San Diego, CA, USA), and Asinex (Moscow, Russia) chemical libraries. To screen these chemicals, 1×10^4^ NF-κB reporter cells were plated in each well of a 384-well plastic cell culture plate in 40 µl DMEM media containing 10% FBS. After incubation overnight, 0.1 µl of the compounds in DMSO and 10 µl lipopolysaccharides (LPS; in DMEM) were added to each well to a final concentration of 10 µM compound and 100 ng/ml LPS. Equal amounts of DMSO and parthenolide were used as negative and positive controls, respectively. To assess the sensitivity and robustness of the cell-based assay before performing HTS, we confirmed dose-response profiles using the positive control parthenolide (Sigma-Aldrich, St. Louis, MO, USA) in the HTS format. Optimization and miniaturization of HTS were performed to achieve 384-well plate formats. After incubation for 8 h with library compounds, lysis buffer containing luciferin was added and the luciferase activity was determined with a luminometer. Compounds that inhibited expression of the reporter gene more than 80% at a concentration of 10 µM were considered to be primary hits. A second HTS was performed using p53 reporter cells in the same manner. Compounds that induced expression of the reporter more than 3-fold at a concentration of 10 µM and showed a clear dose response of the p53 and NF-κB reporters were selected as final hits.

### Western blot analyses

Cellular extracts was prepared as previously described [58]. Proteins were separated using 4–12% gradient SDS-PAGE (Invitrogen) and transferred to nitrocellulose membranes (Bio-Rad). The membranes were blocked with 5% nonfat milk and probed with p53, phosphor-p53 (Ser 6, 9, 15, 20, 37, 46, and 392), phosphor-NF-κB-p65 (Ser536), actin antibodies (Cell Signaling Technology, Boston, MA, USA) and phospho-histone H2AX-PSer139 (Sigma, Saint Louis, USA). The membranes were incubated with horseradish peroxidase-conjugated anti-mouse IgG or anti-rabbit IgG (Sigma-Aldrich) and visualized using the ECL system (Amersham ECL Plus™, USA).

### ELISA assays

Interleukin 6 (IL-6) and monocyte chemotactic protein-1 (MCP-1) levels in the supernatants of RAW 264.7 cells were determined using the mouse IL-6 and MCP-1 Quantikine ELISA kits (R&D Systems, Minneapolis, MN, USA) according to the manufacturer's instructions. Three independent experiments were done, each in triplicate.

### RNA extraction and quantitative reverse transcription-polymerase chain reaction (qRT-PCR)

RNA was isolated using RNeasy kit (Qiagen, Valencia, CA, USA) according to manufacturer's instructions and quantified using spectrophotometer. Relative levels of mRNA were quantified with real-time qPCR using fluorescence TaqMan technology. cDNA was synthesized from 3000 ng total RNA with reverse transcription reagents (Applied Biosystems, Carlsbad CA, USA) according to the manufacturer's instructions. Inventoried PCR primers for human DDIT3 (Hs99999172_m1), DDIT4 (Hs01111686_g1), GADD45 (Hs00169255_m1), SESN2 (Hs00230241_m1), PCNA (Hs00427214_g1), CCND3 (Hs01017690_g1), CDK1 (Hs00938777_m1), and 18S (Hs99999901_s1) were purchased from Applied Biosystems (Carlsbad, CA, USA). 18S RNA was used as an endogenous control. The qPCR was performed using the 7500 real-time PCR system and Taqman universal PCR master mix (Applied Biosystems) according to manufacturer's protocol. Reactions (25 µl) were incubated at 50°C for 2 min, 95°C for 10 sec, followed by 40 cycles of 15 sec at 95°C and 1 min at 60°C. Each sample was analyzed in triplicate. The comparative Ct method was used for relative quantification of gene expression.

### Agar colony formation in A549 human lung cancer cells

Anchorage-independent growth was assayed by the ability to grow in soft agar. The bottom agar was composed of media containing serum and 0.7% agar in a 6-well culture dish. Cells (4×10^3^) were re-suspended in media containing serum and 0.4% agar and plated on top of the bottom agar. Colonies were stained with 0.04% crystal violet acetate and counted after 2 weeks incubation using a vertical microscope.

### Cell viability assays

Cell viability after compound treatment was measured using a luciferase-coupled ATP quantitation assay (CellTiter-Glo viability assay, Promega) in HCT116, A549, B16F10, Jurkat, LLC, H460, H2009, SW480, SH-SY5Y, and C6 cells. Cells (1×10^4^) were plated in a 96-well plate. The cells were incubated for 24 h at 37°C, followed by the addition of the compounds. The assay plates were further incubated for 48 h at 37°C. The IC_50_ was calculated using GraphPad PRISM Software (San Diego, CA, USA). Statistical analyses were performed with a Student's t-test or one-way ANOVA. Differences were considered significant at p<0.05.

### LLC and B16F10 allograft model

B16F10 and LLC cells (1×10^7^) in PBS were inoculated under the right flank of 7-week-old C57/BL6 mice. When tumors reached 5 mm in diameter, daily intraperitoneal administrations of QC (Sigma-Aldrich, 30 mg/kg) or N-2 (1–3 mg/kg) were started. Fourteen to fifteen days after implantation, we measured the tumor size with calipers and calculated tumor volume as (length×width×height). All comparisons were made using an unpaired Student's t test for samples with unequal variance. Differences were considered statistically significant at p<0.05.

All experiments are in accordance with the Korean law on animal care guidelines (8282-13, revised 2007.1.26) and the guidelines set by the SK Biopharmaceuticals Animal Research Policies Committee. All the animal procedures were approved by the institutional animal care and use committee (IACUC) of SK Biopharmaceuticals Co., Ltd (SKLS IACUC-2010-002).

### Global gene expression profiling

We hybridized RNA isolates on microarray Affymetrix GeneChip® Human Gene 1.0 ST Chips by comparing control and compound N-2. After treating A549 cells for 12 h with 1 µM of N-2, global gene expression was profiled following standard protocols. For the identification of differentially expressed genes (DEG), the results were analyzed using Robust Multi-Array Average normalization. We obtained DEG lists of up- and down-regulations with greater than 2-fold changes in expression pattern. Using the DEG lists, we evaluate a molecular interaction network. Protein-protein interaction network is represented as an undirected graph in which nodes indicate DEGs and edges indicate interactions from the MIMI (http://mimi.ncibi.org/MimiWeb/main-page/jsp) database. The visualized network of DEG is generated in Cytoscape, an open source bioinformatics software.

## Supporting Information

Figure S1 **Correlation between NF-κB inhibition potential and p53 fold induction by 9AA derivatives (Group D compounds).** (**A**) Structure: activity relationship table. (**B**) Regression curve between p53 activation and NF-κB inhibition by 9AA derivatives. The results shown are the average of three experiments; bars indicate standard deviation.
(TIF)

Figure S2 **DNA damage signalling is triggered by N-2 treatment in A549 cells.** (**A**) Western blotting analyses of γ-H2AX-PSer^139^. A549 cells were treated with 2 µM N-2 for various time or treated with 1 µM Dox for 16 h. Phosphorylated γ-H2AX levels were analyzed by Western blotting. (**B**) Immunofluorescent staining of γ-H2AX in A549 cells. A549 cells were treated with 2 µM N-2, 1 µM Dox and 5 µM 9AA for 12 h. Cells were incubated with polyclonal H2AX-PSer139 antibody followed by secondary FITC-conjugated anti-rabbit antibody (green). The nucleus was visualized with DAPI (blue). Cells were visualized on a fluorescence microscope at 200× magnification.
(TIF)

Figure S3 **N-2-induced production of ROS in A549 cells.** Generation of ROS in N-2 treated A549 cells was measured by oxidation of redox-sensitive fluorescence probe DCF-DA (10 µM). A549 cells were treated with 2 µM of N-2, 5 µM of 9AA for 10 h. Cells treated with PMA (50 ng/ml) for 1 h were used as a positive control for ROS production. These cells were then stained with DCF-DA for 30 min and their DCF fluorescence was measured by fluorometer (**A**) and visualized by fluorescence microscopy (**B**). Nuclear DNA was visualized with DAPI. The results shown are mean ± SD of three independent experiments. ^#^ p<0.01 by Student's t-test.
(TIF)

Figure S4 **Effect of N-2 on wild type and p53^−/−^ syngenic HCT116 cells.** (**A**) Comparison of the LD50 concentrations of 9AA, 9AA-1, QC and N-2 in wild type and p53^−/−^ syngenic HCT116 cells. (**B**) ROS production by N-2 treatment in wild type and p53^−/−^ syngenic HCT116 cells. Data are mean ± SD of three independent experiments. ^#^ p<0.01 by paired Student's t-test.
(TIF)

Figure S5 **Similarities and differences of N-2 with other related compounds having similar molecular mechanism.** Three-set Venn diagram illustrates the similarities and differences of each molecule.
(TIF)

Methods S1 (DOC)

Table S1 **The list of DEGs with at least two-fold change by N-2 treatment in A549 cells.**
(DOC)

Table S2 **The list of DEGs used for interaction map.**
(DOC)

Table S3 **The LD_50_ concentrations of tumor cell lines.**
(DOC)
