# Supplementary figures and images for: Anti-Cancer Activity of a Novel Small Molecule Compound That Simultaneously Activates p53 and Inhibits NF-κB Signaling
Source: PLoS One. 2012 Sep 13;7(9):e44259. doi: 10.1371/journal.pone.0044259 (PMC3441512; doi:10.1371/journal.pone.0044259)

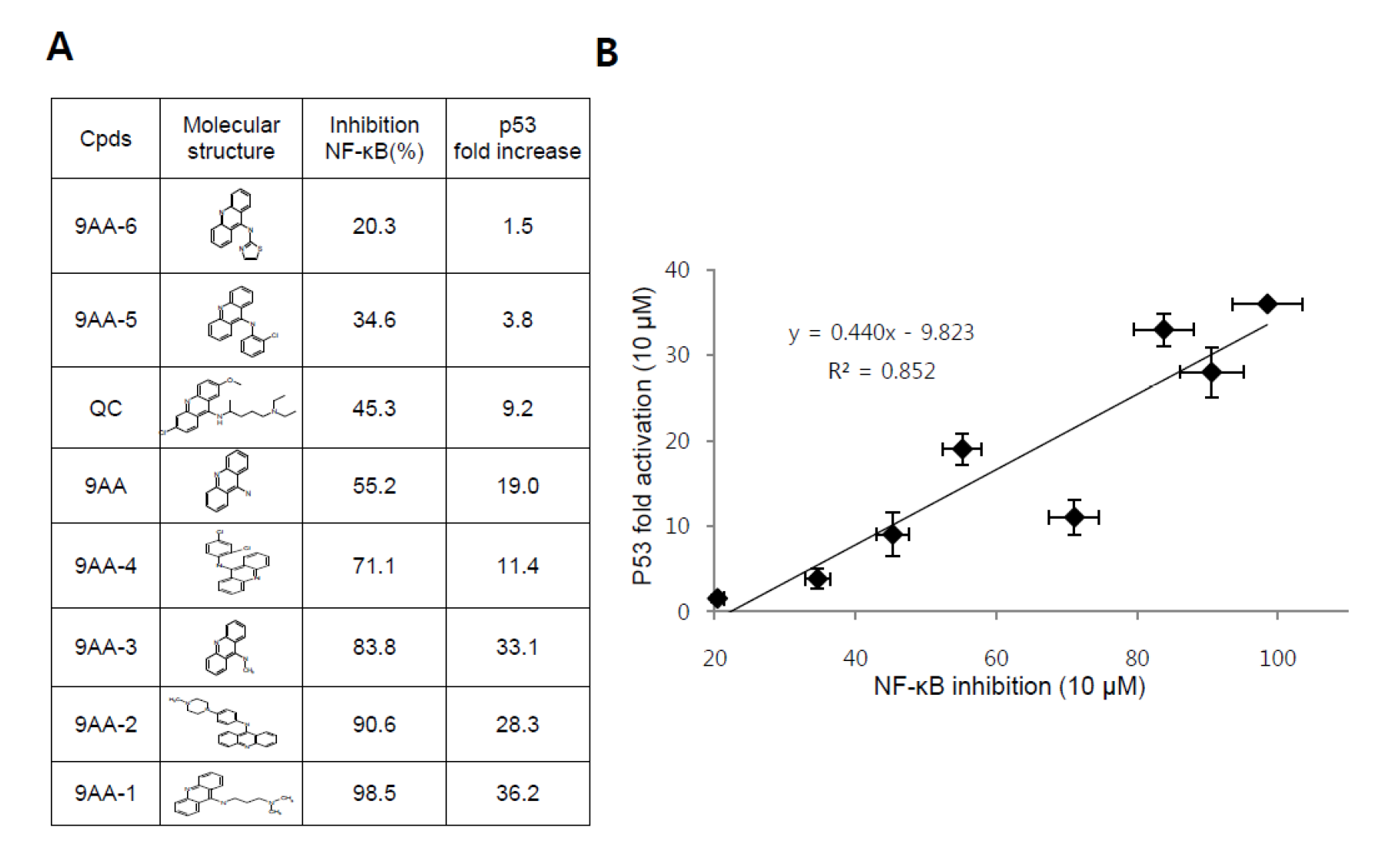

Supplement: Figure S1 — Correlation between NF-κB inhibition potential and p53 fold induction by 9AA derivatives (Group D compounds). (A) Structure: activity relationship table. (B) Regression curve between p53 activation and NF-κB inhibition by 9AA derivatives. The results shown are the average of three experiments; bars indicate standard deviation. (TIF) [file pone.0044259.s001.tif]

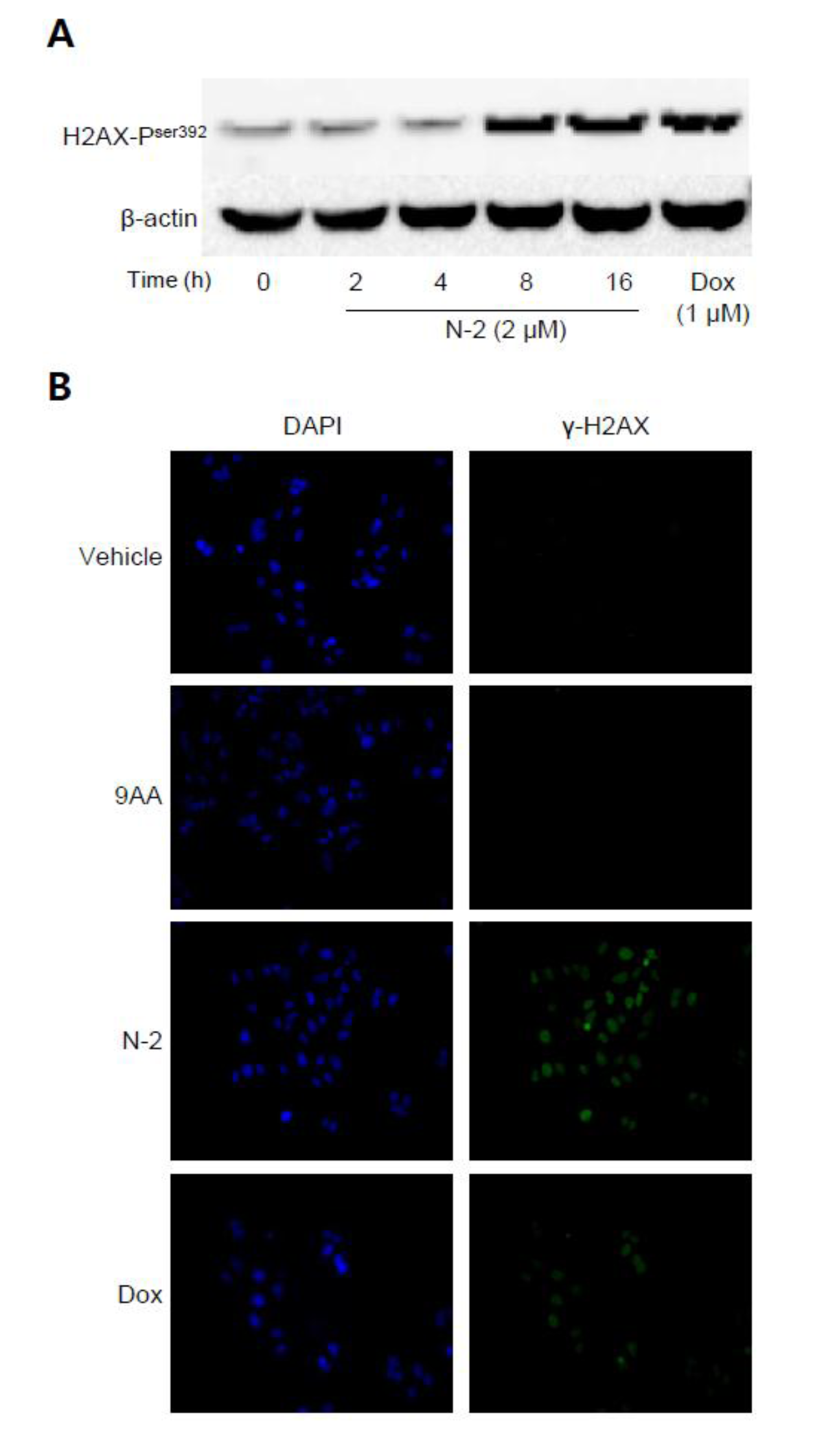

Supplement: Figure S2 — DNA damage signalling is triggered by N-2 treatment in A549 cells. (A) Western blotting analyses of γ-H2AX-PSer139. A549 cells were treated with 2 µM N-2 for various time or treated with 1 µM Dox for 16 h. Phosphorylated γ-H2AX levels were analyzed by Western blotting. (B) Immunofluorescent staining of γ-H2AX in A549 cells. A549 cells were treated with 2 µM N-2, 1 µM Dox and 5 µM 9AA for 12 h. Cells were incubated with polyclonal H2AX-PSer139 antibody followed by secondary FITC-conjugated anti-rabbit antibody (green). The nucleus was visualized with DAPI (blue). Cells were visualized on a fluorescence microscope at 200× magnification. (TIF) [file pone.0044259.s002.tif]

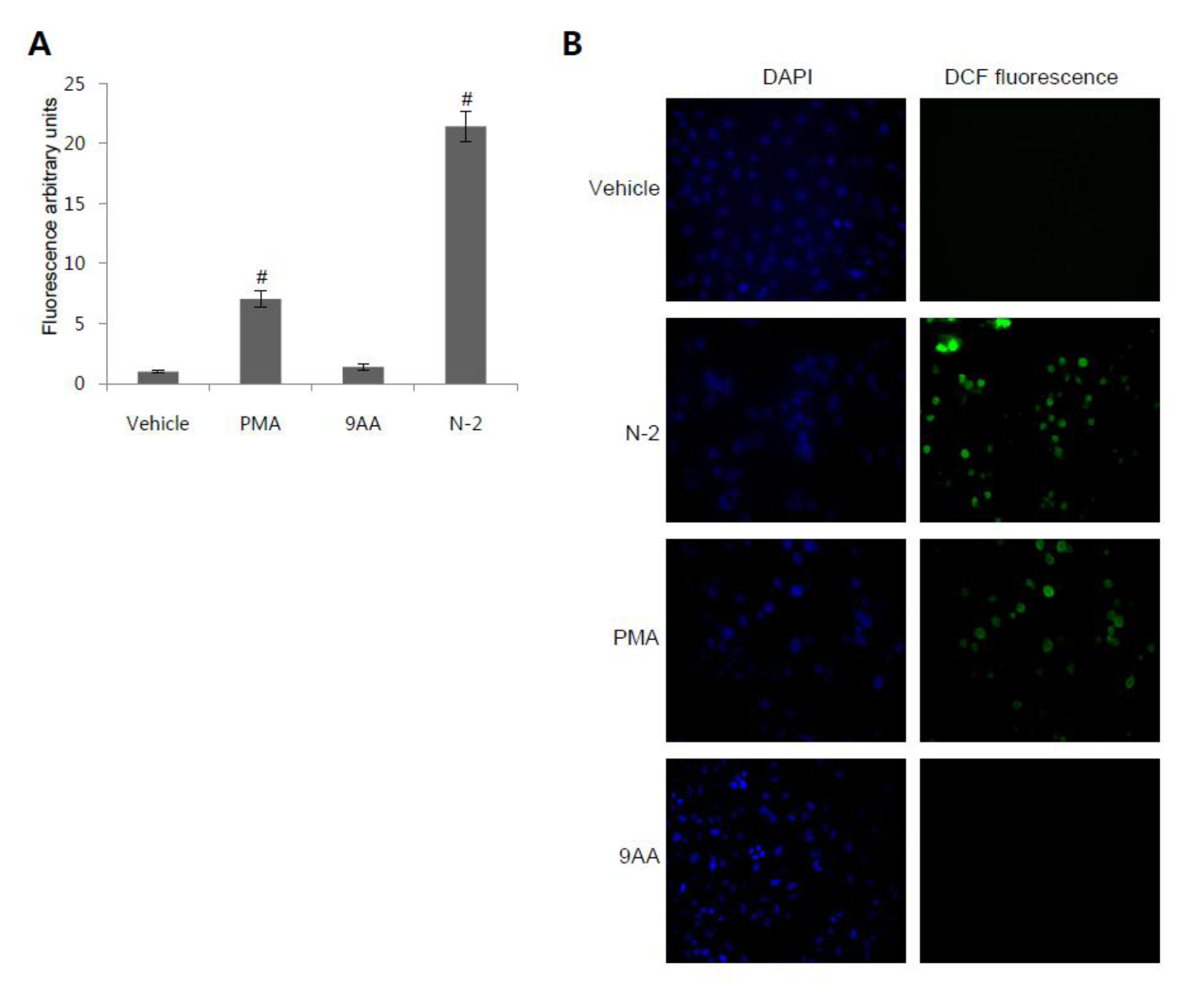

Supplement: Figure S3 — N-2-induced production of ROS in A549 cells. Generation of ROS in N-2 treated A549 cells was measured by oxidation of redox-sensitive fluorescence probe DCF-DA (10 µM). A549 cells were treated with 2 µM of N-2, 5 µM of 9AA for 10 h. Cells treated with PMA (50 ng/ml) for 1 h were used as a positive control for ROS production. These cells were then stained with DCF-DA for 30 min and their DCF fluorescence was measured by fluorometer (A) and visualized by fluorescence microscopy (B). Nuclear DNA was visualized with DAPI. The results shown are mean ± SD of three independent experiments. # p<0.01 by Student's t-test. (TIF) [file pone.0044259.s003.tif]

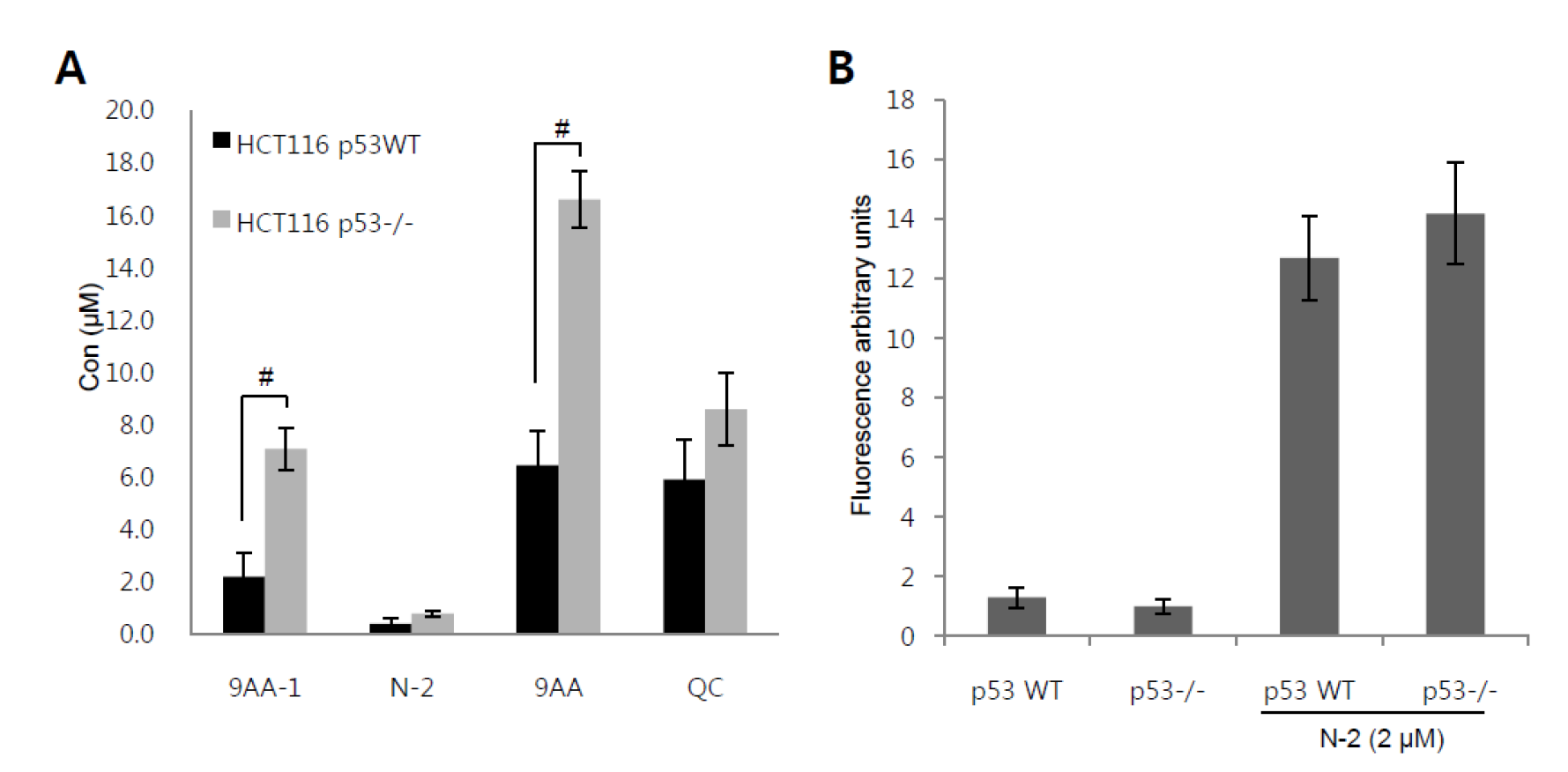

Supplement: Figure S4 — Effect of N-2 on wild type and p53−/− syngenic HCT116 cells. (A) Comparison of the LD50 concentrations of 9AA, 9AA-1, QC and N-2 in wild type and p53−/− syngenic HCT116 cells. (B) ROS production by N-2 treatment in wild type and p53−/− syngenic HCT116 cells. Data are mean ± SD of three independent experiments. # p<0.01 by paired Student's t-test. (TIF) [file pone.0044259.s004.tif]

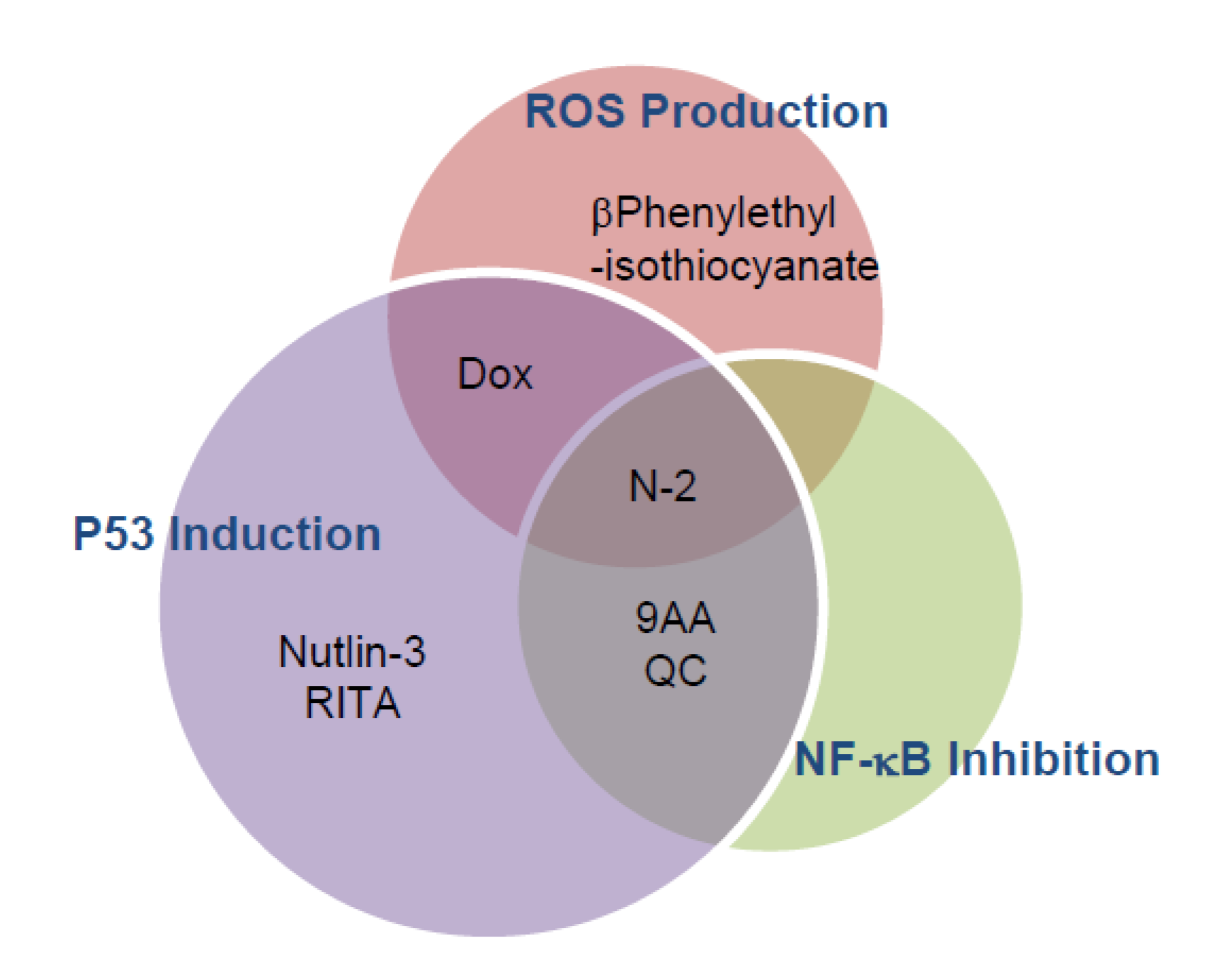

Supplement: Figure S5 — Similarities and differences of N-2 with other related compounds having similar molecular mechanism. Three-set Venn diagram illustrates the similarities and differences of each molecule. (TIF) [file pone.0044259.s005.tif]
