# Supplementary material for: Anti-Cancer Activity of a Novel Small Molecule Compound That Simultaneously Activates p53 and Inhibits NF-κB Signaling
Source: PLoS One. 2012 Sep 13;7(9):e44259. doi: 10.1371/journal.pone.0044259 (PMC3441512; doi:10.1371/journal.pone.0044259)
Supplement: Methods S1 — (DOC) [file pone.0044259.s006.doc]

**Methods S1**

**γ-H2AX staining**

Anti-phospho-Histone H2AX-PSer139 (Sigma, Saint Louis, USA) antibody was used for measuring DNA double strand breaks. A549 cells were plated onto glass coverslips in a 6-well plate and cultured overnight. Cells were then treated with N-2 (2 μM), 9AA (5 μM) and Dox (1 μM) for 12 h. After treatment, cells were washed with PBS, fixed in 4% paraformaldehyde in PBS buffer, permeabilized by 0.3% Triton-X100 and blocked in 4% bovine serum albumin for 1h. The cells were stained overnight with primary anti-γH2AX antibody (1:500 dilution ratios). After overnight incubation, the cells were washed three times with PBS/BSA and incubated for 1 h with anti-rabbit secondary antibody conjugated with FITC (Sigma, Saint Louis, USA) at 1:200 dilution. The cells were washed and counterstained with DAPI (Sigma) before examining the slides. The morphology of cells were observed and captured by fluorescence microscope (Carl Zeiss Axiovert 200) at excitation wavelength of 490 nm for FITC (green) and 350 nm for DAPI (blue).

**Detection of intracellular ROS levels.**

Generation of ROS was assessed by an oxidation-sensitive fluorescent probe 2′,7′-dichlorofluorescein diacetate (DCF-DA, Sigma, Saint Louis, USA). After treatment of cells with N-2 (2 μM) or 9AA (5 μM) for 10 h, attached cells were washed with PBS and then loaded for 30 min with 10 μM of DCF-DA in 2 ml of culture medium. The cells were again washed three times with PBS to remove the extracellular dye, harvested and lysed in 0.1 M Tris containing 10% SDS buffer. The lysates were sonicated for 5 sec three times to reduce the viscosity of samples. The supernatants (100 μl) were assayed for DCF fluorescence with an excitation wavelength of 485 nm and an emission wavelength of 535 nm using fluorometer (Victor, PerkinElmer, USA). The DCF fluorescence was normalized by the protein concentration of individual extracts. Intracellular ROS levels in A549 cells were also determined by fluorescence microscopy after staining with the DCF-DA. Nuclear DNA was visualized by staining with DAPI
