# Supplementary material for: Anti-Cancer Activity of a Novel Small Molecule Compound That Simultaneously Activates p53 and Inhibits NF-κB Signaling
Source: PLoS One. 2012 Sep 13;7(9):e44259. doi: 10.1371/journal.pone.0044259 (PMC3441512; doi:10.1371/journal.pone.0044259)
Supplement: Table S1 — The list of DEGs with at least two-fold change by N-2 treatment in A549 cells. (DOC) [file pone.0044259.s007.doc]

**Table S1.**The list of DEGs with at least two-fold change by N-2 treatment in A549 cells.

1. Up-regulated genes

| **log2 ratio** | **Gene Accession** | | **Gene Symbol** | | **Gene Description** |
| --- | --- | --- | --- | --- | --- |
| 1.57741 | NM_031459 | SESN2 | | sestrin 2 | |
| 1.57411 | NM_005504 | BCAT1 | | branched chain aminotransferase 1, cytosolic | |
| 1.49389 | NM_014398 | LAMP3 | | lysosomal-associated membrane protein 3 | |
| 1.4588 | NM_001042483 | NUPR1 | | nuclear protein, transcriptional regulator, 1 | |
| 1.41971 | NM_001924 | GADD45A | | growth arrest and DNA-damage-inducible, alpha | |
| 1.35929 | NM_001040619 | ATF3 | | activating transcription factor 3 | |
| 1.35559 | NM_001946 | DUSP6 | | dual specificity phosphatase 6 | |
| 1.30343 | NM_002037 | FYN | | FYN oncogene related to SRC, FGR, YES | |
| 1.29905 | NM_175839 | SMOX | | spermine oxidase | |
| 1.29442 | NM_025130 | HKDC1 | | hexokinase domain containing 1 | |
| 1.21244 | NM_019058 | DDIT4 | | DNA-damage-inducible transcript 4 | |
| 1.19651 | NM_001085481 | MAP1LC3B2 | | microtubule-associated protein 1 light chain 3 beta 2 | |
| 1.11659 | NM_152405 | JMY | | junction mediating and regulatory protein, p53 cofactor | |
| 1.8467 | NM_004083 | DDIT3 | | DNA-damage-inducible transcript 3 | |

1. Down-regulated genes

| **log2 ratio** | **Gene Accession** | **Gene Symbol** | **Gene Description** |
| --- | --- | --- | --- |
| -1.00180 | NM_014791 | MELK | maternal embryonic leucine zipper kinase |
| -1.00314 | NM_002592 | PCNA | proliferating cell nuclear antigen |
| -1.01519 | NM_152562 | CDCA2 | cell division cycle associated 2 |
| -1.02129 | NM_024094 | DSCC1 | defective in sister chromatid cohesion 1 homolog |
| -1.02202 | NM_031299 | CDCA3 | cell division cycle associated 3 |
| -1.02935 | AF284753 | UIMC1 | ubiquitin interaction motif containing 1 |
| -1.03257 | NM_181578 | RFC5 | replication factor C (activator 1) 5, 36.5kDa |
| -1.03666 | NM_001761 | CCNF | cyclin F |
| -1.05554 | NM_004217 | AURKB | aurora kinase B |
| -1.05835 | NM_016343 | CENPF | centromere protein F, 350/400ka (mitosin) |
| -1.06912 | NM_003533 | HIST1H3I | histone cluster 1, H3i |
| -1.07040 | NM_001145316 | DSN1 | DSN1, kinetochore complex component, homolog |
| -1.07863 | NM_080668 | CDCA5 | cell division cycle associated 5 |
| -1.10504 | NM_002358 | MAD2L1 | MAD2 mitotic arrest deficient-like 1 (yeast) |
| -1.12917 | NM_006739 | MCM5 | minichromosome maintenance complex component 5 |
| -1.15963 | NM_018136 | ASPM | abnormal spindle homolog, microcephaly associated |
| -1.16399 | NM_016448 | DTL | denticleless homolog |
| -1.17095 | NM_002263 | KIFC1 | kinesin family member C1 |
| -1.17277 | NM_021067 | GINS1 | GINS complex subunit 1 (Psf1 homolog) |
| -1.17637 | NM_020937 | FANCM | Fanconi anemia, complementation group M |
| -1.17844 | NM_004111 | FEN1 | flap structure-specific endonuclease 1 |
| -1.18085 | NM_002689 | POLA2 | polymerase (DNA directed), alpha 2 (70kD subunit) |
| -1.18403 | NM_005914 | MCM4 | minichromosome maintenance complex component 4 |
| -1.18800 | NM_001130862 | RAD51AP1 | RAD51 associated protein 1 |
| -1.19346 | NM_017760 | NCAPG2 | non-SMC condensin II complex, subunit G2 |
| -1.20372 | NM_016195 | KIF20B | kinesin family member 20B |
| -1.21818 | NM_022111 | CLSPN | claspin homolog |
| -1.21945 | NM_022346 | NCAPG | non-SMC condensin I complex, subunit G |
| -1.22039 | NM_199420 | POLQ | polymerase (DNA directed), theta |
| -1.22089 | NM_182513 | SPC24 | SPC24, kinetochore complex component, homolog |
| -1.22376 | NM_003258 | TK1 | thymidine kinase 1, soluble |
| -1.22938 | NM_015341 | NCAPH | non-SMC condensin I complex, subunit H |
| -1.23452 | NM_032997 | ZWINT | ZW10 interactor |
| -1.24177 | NM_016095 | GINS2 | GINS complex subunit 2 (Psf2 homolog) |
| -1.24341 | NM_006328 | RBM14 | RNA binding motif protein 14 |
| -1.24625 | NM_014762 | DHCR24 | 24-dehydrocholesterol reductase |
| -1.25138 | NM_013230 | CD24 | CD24 molecule |
| -1.25480 | NM_024857 | ATAD5 | ATPase family, AAA domain containing 5 |
| -1.26074 | NM_004523 | KIF11 | kinesin family member 11 |
| -1.28336 | NM_001017420 | ESCO2 | establishment of cohesion 1 homolog 2 |
| -1.28569 | NM_001786 | CDC2 | cell division cycle 2, G1 to S and G2 to M |
| -1.28644 | NM_003504 | CDC45L | CDC45 cell division cycle 45-like |
| -1.28991 | NM_001211 | BUB1B | budding uninhibited by benzimidazoles 1 homolog beta |
| -1.29635 | NM_002692 | POLE2 | polymerase (DNA directed), epsilon 2 (p59 subunit) |
| -1.30042 | NM_001254 | CDC6 | cell division cycle 6 homolog |
| -1.30055 | NM_001424 | EMP2 | epithelial membrane protein 2 |
| -1.30301 | NM_001018113 | FANCB | Fanconi anemia, complementation group B |
| -1.31094 | NM_002417 | MKI67 | antigen identified by monoclonal antibody Ki-67 |
| -1.32192 | NM_018410 | HJURP | Holliday junction recognition protein |
| -1.36813 | NM_001760 | CCND3 | cyclin D3 |
| -1.37408 | NM_001080449 | DNA2 | DNA replication helicase 2 homolog |
| -1.40924 | NM_130398 | EXO1 | exonuclease 1 |
| -1.41015 | NM_032043 | BRIP1 | BRCA1 interacting protein C-terminal helicase 1 |
| -1.42754 | NM_057749 | CCNE2 | cyclin E2 |
| -1.43866 | NM_018154 | ASF1B | ASF1 anti-silencing function 1 homolog B |
| -1.45251 | NM_024680 | E2F8 | E2F transcription factor 8 |
| -1.48781 | NM_020675 | SPC25 | SPC25, kinetochore complex component, homolog |
| -1.49936 | NM_182751 | MCM10 | minichromosome maintenance complex component 10 |
| -1.53034 | NM_017669 | ERCC6L | excision repair cross-complementing rodent repair deficiency, complementation group 6-like |
| -1.76087 | NM_001034 | RRM2 | ribonucleotide reductase M2 |
