# Supplementary material for: Anti-Cancer Activity of a Novel Small Molecule Compound That Simultaneously Activates p53 and Inhibits NF-κB Signaling
Source: PLoS One. 2012 Sep 13;7(9):e44259. doi: 10.1371/journal.pone.0044259 (PMC3441512; doi:10.1371/journal.pone.0044259)
Supplement: Table S2 — The list of DEGs used for interaction map. (DOC) [file pone.0044259.s008.doc]

**Table S2.**The list of DEGs used for interaction map.

| **log2 ratio** | **Gene Accession** | **Gene Symbol** | **GO Biological Process Term** |
| --- | --- | --- | --- |
|  |  |  |  |
| 1.303427 | NM_002037 | FYN | protein amino acid phosphorylation // calcium ion transport // protein kinase cascade |
| 1.0742 | NM_001001555 | GRB10 | signal transduction // cell-cell signaling // negative regulation of glucose import |
|  |  |  | **Stress response and apoptosis** |
| 1.419708 | NM_001924 | GADD45A | apoptosis // response to DNA damage stimulus // cell cycle arrest // response to stress |
| 1.164816 | NM_005531 | IFI16 | DNA damage response, signal transduction by p53 - apoptosis |
| 1.577409 | NM_031459 | SESN2 | cell cycle arrest // response to oxidative stress |
| 1.359288 | NM_001040619 | ATF3 | regulation of transcription // apoptosis |
| 1.244288 | NM_021158 | TRIB3 | apoptosis // response to stress // |
| 1.846703 | NM_004083 | DDIT3 | response to oxidative stress // ER overload response// positive regulation of apoptosis |
| 1.116589 | NM_152405 | JMY | induction of apoptosis // response to DNA damage stimulus |
|  |  |  | **DNA repair** |
| -1.003141 | NM_002592 | PCNA | regulation of DNA replication // DNA gap filling // mismatch repair |
| -1.084101 | NM_000057 | BLM | double-strand break repair via homologous recombination // DNA repair |
| -1.178444 | NM_004111 | FEN1 | double-strand break repair // DNA replication, removal of RNA primer |
| -1.409242 | NM_130398 | EXO1 | mismatch repair // mismatch repair // mismatch repair // mismatch repair |
| -1.374079 | NM_001080449 | DNA2 | mitochondrial DNA replication // base-excision repair |
| -1.564905 | NM_003513 | HIST1H2AB | nucleosome assembly |
| -1.013905 | NM_000465 | BARD1 | DNA repair // response to DNA damage stimulus // cell cycle arrest |
|  |  |  | **Cell cycle** |
| -1.285694 | NM_001786 | CDC2 | cell cycle // mitosis //cell division |
| -1.203722 | NM_016195 | KIF20B | microtubule-based movement // cell cycle // regulation of mitosis // cell division |
| -1.036658 | NM_001761 | CCNF | re-entry into mitotic cell cycle // cell cycle // mitosis // cell division |
| -1.015172 | NM_012291 | ESPL1 | positive regulation of mitotic metaphase/anaphase transition |
| -1.260736 | NM_004523 | KIF11 | microtubule-based movement // cell cycle // mitotic spindle organization // mitosis |
| -1.300419 | NM_001254 | CDC6 | regulation of cyclin-dependent protein kinase activity // cell cycle // mitosis |
| -1.368131 | NM_001760 | CCND3 | cell cycle // positive regulation of cyclin-dependent protein kinase activity // cell division |
| -1.105043 | NM_002358 | MAD2L1 | cell cycle // mitosis // mitotic cell cycle checkpoint |
| -1.234516 | NM_032997 | ZWINT | cell cycle // mitotic cell cycle checkpoint // cell division |
| -1.070402 | NM_001145316 | DSN1 | cell cycle // chromosome segregation // mitosis // cell division |
| -1.094206 | NM_018492 | PBK | protein amino acid phosphorylation // mitosis |
| -1.28644 | NM_003504 | CDC45L | DNA replication // DNA replication // DNA replication initiation // cell cycle |
|  |  |  | **DNA replication** |
| -1.021286 | NM_024094 | DSCC1 | DNA replication // cell cycle // maintenance of mitotic sister chromatid cohesion |
| -1.223756 | NM_003258 | TK1 | nucleobase, nucleoside, nucleotide and nucleic acid metabolic process // DNA replication |
| -1.760867 | NM_001034 | RRM2 | DNA replication // deoxyribonucleoside diphosphate metabolic process |
| -1.032566 | NM_181578 | RFC5 | DNA replication // DNA repair // nucleotide-excision repair, DNA gap filling |
| -1.180852 | NM_002689 | POLA2 | protein import into nucleus, translocation // DNA replication |
| -1.296349 | NM_002692 | POLE2 | DNA-dependent DNA replication // DNA repair // nucleotide-excision repair |
| -1.218182 | NM_022111 | CLSPN | DNA replication // DNA repair // response to DNA damage stimulus // cell cycle |
| -1.172771 | NM_021067 | GINS1 | inner cell mass cell proliferation // DNA replication |
| -1.241766 | NM_016095 | GINS2 | DNA replication |
| -1.575545 | NM_004153 | ORC1L | DNA replication // DNA replication // DNA replication initiation |
| -1.184031 | NM_005914 | MCM4 | DNA replication // DNA unwinding during replication // DNA replication initiation |
| -1.129169 | NM_006739 | MCM5 | DNA replication // DNA replication // DNA replication initiation |
| -1.499361 | NM_182751 | MCM10 | DNA replication // DNA replication |
