# Supplementary material for: Anti-Cancer Activity of a Novel Small Molecule Compound That Simultaneously Activates p53 and Inhibits NF-κB Signaling
Source: PLoS One. 2012 Sep 13;7(9):e44259. doi: 10.1371/journal.pone.0044259 (PMC3441512; doi:10.1371/journal.pone.0044259)
Supplement: Table S3 — The LD50 concentrations of tumor cell lines. (DOC) [file pone.0044259.s009.doc]

**Table S3. The LD_50_ concentrations of tumor cell lines.**

| **LD_50_(µM)**  **Mean±SD** | **p53 Wild Type** | | | | | | **p53 Mutant** | | | | | **Statistics**  **(WT vs Mut)** |
| --- | --- | --- | --- | --- | --- | --- | --- | --- | --- | --- | --- | --- |
| **Cell line** | **A549** | **H460** | **HCT116** | **C6** | **SH-5Y** | **B16F10** | **H2009** | **SW480** | **Jurkat** | **U937** | **LLC** | **p value**  **(T-test)** |
| **9AA** | **7.4 ± 0.8** | **1.8 ± 0.2** | **6.5 ± 0.5** | **6.8 ± 0.6** | **7.1 ± 0.7** | **10.5 ± 1.0** | **13.1 ± 1.3** | **13.3 ± 0.8** | **0.63 ± 0.04** | **5.1 ± 0.3** | **13.1 ± 1.0** | **0.40** |
| **QC** | **6.9 ± 0.4** | **7.1 ± 0.4** | **5.6 ± 0.3** | **8.5 ± 0.5** | **15.1 ± 1.1** | **12.3 ± 0.9** | **9.8 ± 0.7** | **10.5 ± 0.8** | **6.2 ± 0.3** | **31.5 ± 1.7** | **33.5 ± 1.4** | **0.14** |
| **9AA-1** | **2.2 ± 0.2** | **8.7 ± 0.7** | **2.2 ± 0.1** | **3.2 ± 0.4** | **1.8 ± 0.2** | **8.2 ± 0.5** | **4.6 ± 0.2** | **3.4 ± 0.2** | **1.1 ± 0.1** | **12.3 ± 0.6** | **21.3 ± 1.1** | **0.28** |
| **N-2** | **0.62 ± 0.04** | **1.13 ± 0.05** | **0.53 ± 0.04** | **1.03 ± 0.06** | **0.42 ± 0.04** | **0.6 3± 0.03** | **0.92 ± 0.04** | **1.21 ± 0.05** | **0.24 ± 0.02** | **0.71 ± 0.04** | **0.083 ± 0.003** | **0.72** |

|  |  |  |  |
| --- | --- | --- | --- |
|  |  |  |  |
|  |  |  |  |
|  |  |  |  |
|  |  |  |  |
|  |  |  |  |
|  |  |  |  |
|  |  |  |  |
|  |  |  |  |
|  |  |  |  |
|  |  |  |  |
|  |  |  |  |
|  |  |  |  |
|  |  |  |  |
|  |  |  |  |
|  |  |  |  |
|  |  |  |  |
|  |  |  |  |
|  |  |  |  |
|  |  |  |  |
|  |  |  |  |
|  |  |  |  |
